# Kinetically driven switching and memory phenomena at the interface between a proton-conductive electrolyte and a titanium electrode

**DOI:** 10.1038/srep31691

**Published:** 2016-08-16

**Authors:** Takashi Hibino, Kazuyo Kobayashi, Masahiro Nagao

**Affiliations:** 1 Graduate School of Environmental Studies, Nagoya University, Nagoya 464-8601, Japan

## Abstract

Numerous studies have examined the switching properties of semi- or ion-conductors and isolators; however, most of these have focused on the ohmic resistance characteristics. Here, we report a new type of polarity-dependent switching phenomenon obtained for electrical devices with the configuration: metal working electrode│Si_0.97_Al_0.03_H_0.03_P_2_O_7_-polytetrafluoroethylene composite electrolyte│Pt/C counter electrode. The counter electrode is reversibly active for the water vapor oxidation and evolution reactions. The composite electrolyte exhibits high withstanding voltage capability in the bias voltage range of ±7 V. When titanium was employed as the working electrode, the anodic polarization resistance was approximately two orders of magnitude greater than the cathodic polarization resistance. The ohmic resistance of the device was almost unchanged, regardless of the bias voltage polarity. Moreover, kinetically induced high-resistance/low-resistance states could be cyclically switched through positive/negative bias voltage pulses, and these states were also confirmed to be memorized at open circuit.

A variety of metal oxides and sulfides, including perovskite-type oxides^1^,^2^,^3^,^4^,^5^, transition metal oxides^6^,^7^,^8^,^9^,^10^, and solid electrolytes^11^,^12^,^13^,^14^,^15^,^16^, exhibit resistive switching characteristics by configuring two electrodes on the same or opposite surfaces of these materials, followed by alternating the amplitude or polarity of voltage. The resultant switching between a high-resistance state (HRS) and low-resistance state (LRS) can be exploited as nonvolatile memory. Although the mechanism for resistive switching has not yet been clarified, the switching observed for solid electrolytes is relatively better understood than that for perovskite-type oxides and transition metal oxides^17^,^18^,^19^,^20^,^21^. The switching in solid electrolytes is attributable to the formation and decomposition of conductive filaments in the electrolyte bulk. Metal ions are electrochemically reduced to the metallic state, so that the filaments are formed as continuous charge pathways between the two electrodes. The filaments are then reoxidized to the metal ions by the application of an opposite voltage. Conductive filaments have been developed in both cation- and anion-type electrolytes, and in the latter, oxygen vacancies act as mediators for charge redistribution^22^,^23^,^24^. In either case, the change in ohmic resistance is responsible for the switching phenomena with these solid electrolytes.

The overall internal resistance of solid electrolyte cells is the sum of the ohmic and polarization resistances. The polarization resistance is determined by the charge-transfer and diffusion kinetics that occur at the electrolyte-electrode interface under polarization^25^. These processes are essentially reversible in the anodic and cathodic cycles, but this reversibility is nullified by the compositional or structural change of the interface under alternate polarization conditions. There is a possibility that the gap in polarization resistance between the two reactions is sufficiently large, and that the transition from HRS to LRS and vice versa are performed repeatedly by SET and RESET operations, respectively. Josberger *et al*. reported that the PdH_x_│Nafion│PdH_x_ electrochemical cell exhibits synapse-like memory behavior based on the depletion of hydrogen from the PdH_x_ electrode after successive anodic voltage pulses^26^. In this study, we propose another type of kinetic switching phenomenon that is ascribed to the difference in catalytic activity for the electrode reaction between two electrode materials. While platinum usually acts as an active electrode for both the water vapor oxidation and evolution reactions, gold is assumed to be less active for such reactions. On the other hand, titanium, chromium, and aluminum are also expected to be more inert for the water vapor oxidation reaction than for the water vapor evolution reaction, due to the growth of nanometer-thin surface oxides (so-called passivation films) and their corrosion at more positive bias voltages, in contrast to their immunity from such passivation barriers at negative bias voltages. In particular, passivation films can easily regenerate after rupture, which enables reversible switching. Furthermore, the kinetics of the water vapor oxidation reaction may be reduced by using a plate form of metal electrode, which more limits the diffusion of water vapor to the electrode, compared to the mesh-form metal electrode. The validity of these approaches was examined by the fabrication of an electrochemical cell consisting of various metal working electrodes, a Pt/C counter electrode, and a proton-conducting solid electrolyte.

## Results

Si_0.97_Al_0.03_H_0.03_P_2_O_7_ (SAHPO) was selected as the electrolyte material because proton conduction in this material is based on a hopping mechanism rather than the Grotthuss mechanism^27^,^28^; therefore, it does not require excessive humidification, unlike sulfonated polymers. The electrolyte membrane was prepared by simply mixing SAHPO powder with polytetrafluoroethylene (PTFE) powder, followed by cold-rolling the mixture to a thickness of 250 μm. The physicochemical characteristics of the composite membrane are summarized in Table S1 (ESI). The proton conductivity of the composite membrane was 5.92 × 10^−4^ S cm^−1^, which gave an area-specific ohmic resistance of 42.3 Ω cm^2^. The total acid content determined according to the method suggested by Boehm^29^ was 0.07 mEq g^−1^, which corresponds to a pH of approximately 4. The H_2_ and O_2_ penetration rates reached 5.94 and 0.61 mmol m^−2^ s^−1^, respectively, due to physical gas leakage through the membrane.

**Figure 1:**
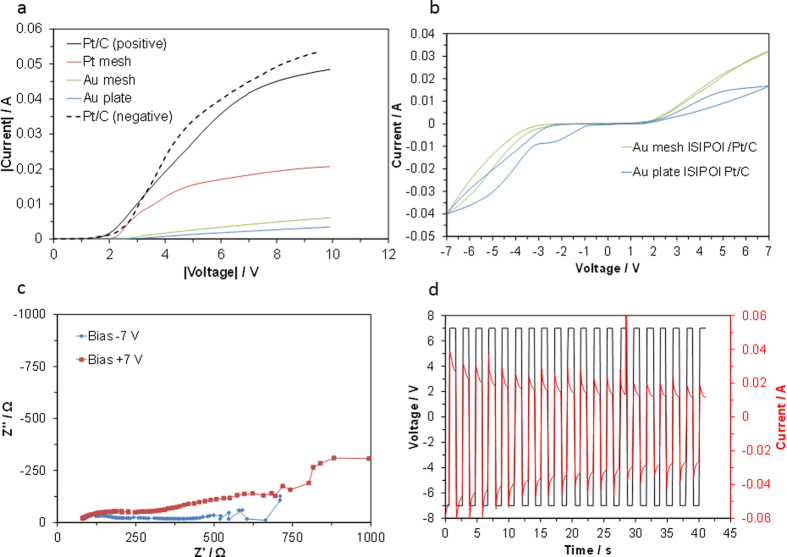
(**a**) LSV curves for various electrodes with composite electrolyte membrane. (**b**) CV profiles for a Au plate│composite electrolyte│Pt/C device (with data for a Au mesh electrode included for comparison), (**c**) impedance spectra at bias voltages of ±7 V, and (**d**) transient changes in the bias voltage and current signals measured at 1 s intervals.

Linear sweep voltammetry (LSV) curves for various electrodes with the composite electrolyte membrane were recorded in atmospheric air (Fig. 1a). The current started to flow between 1.5 and 3.0 V, depending on the electrode employed, and then almost linearly increased with the voltage up to approximately 7 V. The LSV results also indicate that the current at each voltage increased with the electrode selection in the order of Pt/C, Pt mesh, Au mesh, and Au plate, which is the same as that predicted according to the activity for the water vapor oxidation and evolution reactions^30^,^31^. Thus, it is reasonable to conclude that the withstanding voltage of the composite membrane is at least 7 V, which is supported by the polarity symmetric behavior of the Pt/C electrode, as shown by the solid line (positive voltage) and dotted line (negative voltage) in Fig. 1a. A switching device could thus be constructed with the most active Pt/C electrode and the least active Au plate electrode among the tested electrodes.

Cyclic voltammetry (CV) profiles for such a device were recorded over the voltage range of ±7 V in two-electrode mode, and the Au mesh electrode was also compared with the Au plate electrode (Fig. 1b). The CV profiles showed two distinct features between the two Au electrodes; a relatively large reduction peak was observed for the Au plate before the onset of the water vapor evolution reaction, which will be discussed later, and the current attributable to the water vapor oxidation reaction was lower for the Au plate than for the Au mesh electrode. The latter difference is due to suppression of water vapor supply to the electrode/electrolyte interface by the dense plate; however, this effect gave a small cathodic to anodic current ratio of approximately 2. Similar effects were observed for impedance spectra measured under anodic and cathodic polarization (Fig. 1c) and for the switching characteristics in the pulse mode (Fig. 1d). (The decrease in current with the time observed in Fig. 1d is probably ascribed to the oxidation of Au for the working electrode and to the corrosion of carbon for the counter electrode by the application of the positive and negative voltage pulses, respectively, to the device, as described later.) These results indicate that the performance of this device is much lower than those of other types of switching devices. However, the results also suggest that the plate-form electrode is more favorable than mesh- or powder-form electrodes for the appearance of switching.

**Figure 2:**
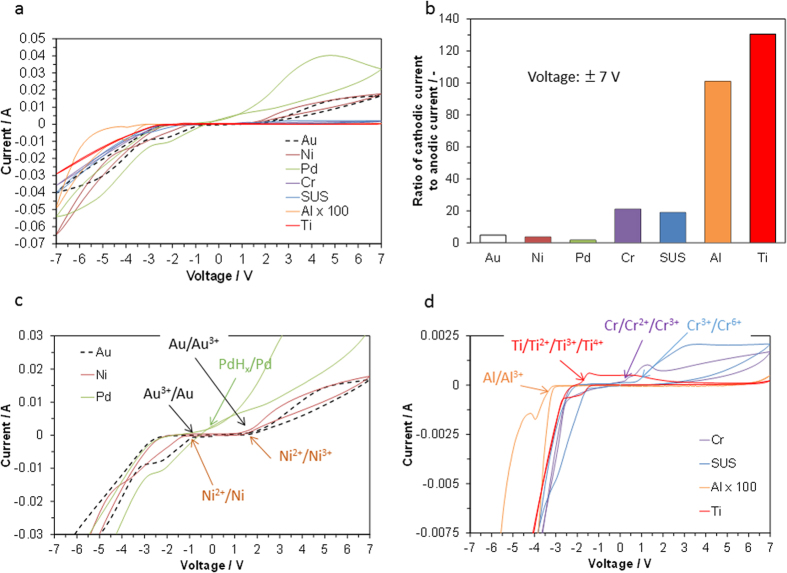
Electrode properties of metal (Au, Ni, Pd, Cr, SUS, Al, and Ti) plates│composite electrolyte│Pt/C devices; (**a**) CV profiles, (**b**) cathodic/anodic current ratios, and (**c**) enlarged CV profiles for Au, Ni, and Pd and (**d**) for Cr, SUS, Al, and Ti.

Various metal plates were examined as working electrodes in place of the Au plate, and CV profiles were measured with the SAHPO electrolyte and Pt/C counter electrode (Fig. 2a). Reversal of the current polarity occurred for all the tested electrodes between approximately −3 and +3 V. All tested electrodes, except Pd, had asymmetric current distributions in the CV curve across the zero-current axis. The cathodic/anodic current ratio was significantly affected by the metal species (Fig. 2b), giving ratios that were one or two orders of magnitude greater for Cr (and stainless steel (SUS)), Al, and Ti than for Au, Ni, and Pd. To better understand these results, the CV profiles were categorized into two groups according to the current ratio (Fig. 2c,d). In the group with small current ratios, Pd showed no voltage gap between the onset voltages of the water vapor oxidation and evolution reactions, which suggests that reversible hydrogen storage and release occur mainly under cathodic and anodic polarization, respectively. Both Au and Ni presented metal ion/metal reduction peaks under cathodic polarization and metal or low-valent metal ion/high-valent metal ion oxidation peaks under anodic polarization, in addition to the current caused by the water vapor reduction and evolution reactions. Similarly, in the group with large current ratios, some peaks attributable to metal redox reactions were observed in the CV profiles; however, the peak current values for Cr (and SUS), Al, and Ti were significantly lower than those for Au and Ni, which reflects the large difference in the oxidation depth of the metals in these two groups. This difference results from the formation, or lack thereof, of a surface oxide barrier under anodic polarization, and this will be verified in the next section.

**Figure 3:**
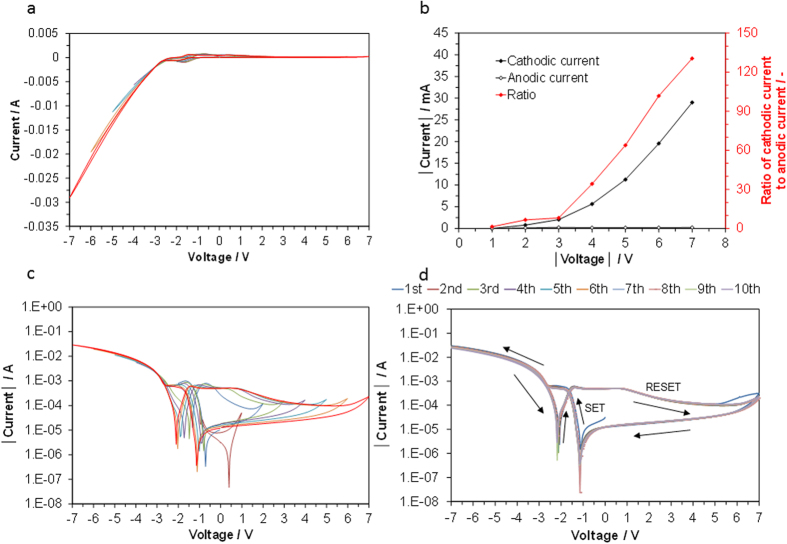
Electrode properties of the Ti plate│composite electrolyte│Pt/C device; (**a**) CV profiles at ±1 V (brawn), ±2 V (blue), ±3 V (green), ±4 V (violet), ±5 V (sky-blue), ±6 V (orange), and ±7 V (red), (**b**) cathodic and the anodic currents and the cathodic/anodic current ratio as a function of voltage, and (**c**) logarithmic plots of the CV profiles in a), and d) CV profiles for the first 10 cycles.

Based on the apparently superior properties of Ti, this material was the focus of subsequent experiments. The CV profiles for the device were obtained in the voltage ranges of ±1 and ±7 V (Fig. 3a). Under cathodic polarization, the outward and return currents were almost proportional to the voltage in a similar manner. In contrast, under anodic polarization, the outward current slightly decreased with the voltage from 0 to +3 V and then reached almost zero with further polarization, while the return current was maintained at zero level for all tested voltages. As a result, the cathodic/anodic current ratio abruptly increased over the voltage range of 3–7 V (Fig. 3b). To gain additional insight into these results, the CV profiles shown in Fig. 3a were replotted as the logarithms of the absolute currents. Two strongly asymmetric hysteresis loops characterized by the presence of two current minima appeared from −3 to +7 V (Fig. 3c), the behavior of which is different from those observed for filament- and interface-type resistive switching^17^,^18^,^19^,^20^,^21^. Moreover, X-ray diffraction (XRD) and scanning electron microscopy (SEM) observations before and after the measurements showed no change in the crystalline structure and morphology of the electrode/electrolyte interface (Fig. S1 in ESI). Thus, the origin of the hysteretic behavior for the present device could be simply ascribed to the occurrence of redox reactions between Ti and TiO_x_, i.e., the reduction of surface oxides to the metallic state under cathodic polarization and vice versa under anodic polarization. It is noteworthy that the hysteresis redox curves remained almost unchanged after 10 scans (Fig. 3d).

**Figure 4:**
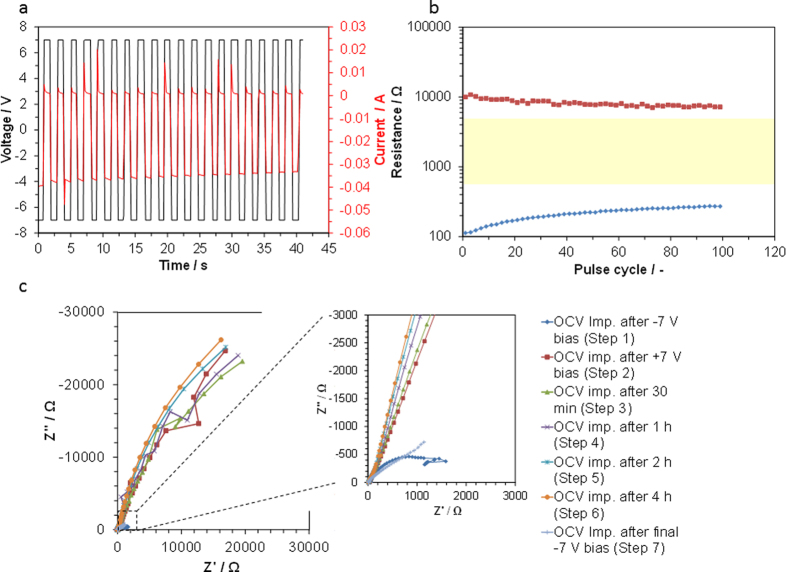
Switching and memory characteristics of the Ti plate│composite electrolyte│Pt/C device. (**a**) transient changes in the bias voltage and current signals measured at 1 s intervals. (**b**) retention behavior in the resistance of two states induced by pulsed voltages for 0.1 s with cycle number. (**c**) impedance spectra at open-circuit voltage after application of a bias voltage.

From the CV profiles (Fig. 3), the voltage pulses for switching operation were determined to be amplitudes of ±7 V. Voltage pulses of −7 V provided high currents from −39.5 to −33.2 mA and reverse voltage pulses depressed the current from +0.8 to +1.0 mA in consecutive operations (Fig. 4a). The resistance of the device changed reversibly between HRS and LRS by alternating pulsed voltages (Fig. 4b), whereby resistance ratios of HRS to LRS initially reached 90 but decreased to 27 after 100 cycles. This degradation is due to carbon corrosion at the Pt/C counter electrode during the water vapor oxidation reaction, which increases the resistance value of the LRS with the cycle number. Carbon corrosion for Pt/C is a well-known phenomenon in electrolysis and fuel cells^32^,^33^, caused by the oxidation of carbon to CO_2_ by water vapor^34^. The retention performance of the device was evaluated by applying a voltage of −7 V to the device for 10 s, followed by recording the impedance at an open circuit voltage of ca. −0.5 V (Step 1), which gave a Z’ value of approximately 1500 Ω at 0.1 Hz (Fig. 4c). A voltage of +7 V was then applied to the device for 10 s and the impedance was recorded at an open circuit voltage of ca. +0.4 V (Step 2), which resulted in impedance with the shape of half a semicircle, where the Z’ value at 0.1 Hz was approximately 20000 Ω. (Equivalent circuit models for the impedance and bode plots in Steps 1 and 2 are provided in Figs S2 and S3, respectively, in ESI.) The impedance was then monitored under open-circuit conditions with intervals (Steps 3–6), and the impedance was almost unchanged during this period. Finally, the impedance was significantly reduced by conducting the same procedure as Step 1 (Step 7), where the amplitude of Z’ and Z” are far nearer those of the impedance observed for Step 1, compared to those of the impedances found for Steps 2–6. Consequently, it is concluded that the present device remains nonvolatile for at least 4 h. However, it should be noted that the overall internal resistances of the device in Steps 1 and 2, which are estimated from the equivalent circuit models (Figs S2 and S3 in ESI), are larger than the resistance values of the LRS and HRS shown in Fig. 4b. This is not unexpected, since no bias voltage was applied to the device in the above steps, the polarization resistances of which are larger compared to those obtained at bias voltages^35^,^36^.

## Discussion

**Figure 5:**
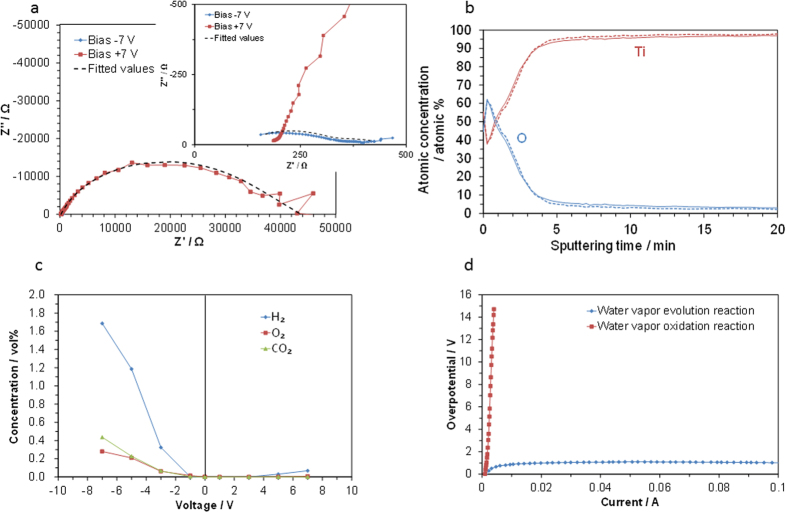
(**a**) Impedance spectra at bias voltages of ±7 V. (**b**) Auger depth profiles of the Ti plate electrode before (solid line) and after (dotted line) electrochemical testing. (**c**) H_2_, O_2_, and CO_2_ concentrations in the effluent gases from the device during cathodic and anodic polarization as a function of current. (**d**) overpotential of the Ti powder electrode for the water vapor evolution and oxidation reactions.

The relationship between the reaction kinetics and passivation film on the Ti surface are discussed here. Nyquist plots of the device showed that the anodic polarization resistance was approximately two orders of magnitude larger than the cathodic polarization resistance (Fig. 5a), which corresponds well with the performance of the device. The equivalent circuit used to interpret the data represents a combination of charge-transfer and gas diffusion impedances in series: the serial resistance R_s_, the charge transfer resistance R_CT_, the constant phase element CPE, and the Warburg short impedance W_S_ (dotted lines of Fig. 5a). The computed R_s_ value, which corresponds to the ohmic resistance, was not significantly dependent on the bias voltage polarity (inset of Fig. 5a).

The compositions and thicknesses of the oxide layer grown on the Ti surface before and after the electrochemical measurements were characterized. Signals from the Ti, O, and C elements appeared in both Auger spectra. The origin of the C signal is a contamination layer. The depth profiles (excluding carbon) revealed that after etching for more than 4 min, the Ti and O concentrations in both the samples became almost constant (Fig. 5b). The thickness of the oxide layers determined from the etching depth was 9 nm for both samples. The lack of change in the oxide layer thickness before and after the measurements indicates that this layer is stable during operation or is retrieved promptly after the operation, which satisfies the terms for a passivation film. In contrast, the thicknesses of the oxide layer for the Au electrode with a low current ratio were as thin as 0.2 and 0.7 nm before and after the electrochemical measurements, respectively, (Fig. S4 in ESI), which indicates the absence of a passive barrier film on the surface of this sample.

To elucidate the reaction kinetics on the Ti surface, the effluent gases from the device were analyzed with argon as the carrier gas rather than atmospheric air because the accuracy of this analysis is not high for water vapor. H_2_, O_2,_ CO_2_, and water vapor were detected during cathodic and anodic polarization. The H_2_, O_2_, and CO_2_ concentrations increased with the voltage, especially under cathodic polarization (Fig. 5c). It should be noted that the device draws a much higher cathodic current than anodic current; therefore, the amounts of produced gases are different for these two conditions. Accordingly, the following reactions are assumed to occur at the working and counter electrodes under cathodic polarization:

Working electrode:


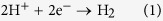


Counter electrode:


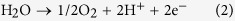



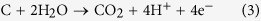


In atmospheric air, Reaction (1) would be replaced by the water vapor evolution reaction:

Working electrode:


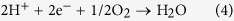


Reactions (2) and (1) correspond to the electrode reactions at the working and counter electrodes, respectively, under anodic polarization. The water vapor oxidation reaction shown by Reaction (2) is also available at the working electrode in atmospheric air.

From these results, it is considered that the water vapor oxidation reaction is more kinetically limited at the Ti electrode than the water vapor evolution reaction, which is closely related to the formation of the passivation film on the Ti surface and the subsequent corrosion under anodic polarization; therefore, the redox potentials of such reactions correspond to the SET and RESET voltages shown in Fig. 3d. However, this limitation also involves two external factors; the exhaustion of the water vapor source at the electrode/electrolyte interface caused by the dense plate, and the poor current collection ability of the passive film-covered electrode, which contributes to an increase in the polarization resistance. To eliminate these factors from the reaction kinetics, the IR-free overpotentials for the water vapor oxidation and evolution reactions were assessed with a titanium powder electrode. The three-electrode method generated a significantly large difference in the polarization resistance for the two reactions without any external factors (Fig. 5d). One possible explanation for the poor water vapor oxidation reaction kinetics is that the occupation of oxygen intermediates on the TiO_2_ surface sites requires a large energy input, which causes the high overpotential for this reaction^37^.

## Conclusions

We have demonstrated kinetically-induced switching and memory effects by cathodic and anodic polarization of a Ti working electrode. In particular, the application of positive (+7 V) and negative (−7 V) bias voltages results in polarization resistance with a difference of approximately two orders of magnitude between the two operations. Reversible switching between the LRS and HRS is always accomplished by redox reactions between metal and metal ions, which correspond to the SET and RESET operations. The origin of the HRS is attributable to the significantly low activity of anodically-polarized Ti for the water vapor oxidation reaction. It was also confirmed that no power input is required to maintain the resistance within a given state. These characteristics are strongly related to the formation and stability of the passivation film grown on the Ti surface.

## Methods

### Materials

The metal (0.1 mm thick, purity 99.5–99.9%, Nilaco) and stainless steel (SUS316, 0.3 mm thick, Hohsen) plates examined were used as working electrodes without any pre-treatment. Commercially available Pt/C brushed on a gas diffusion layer (40 wt% Pt, ElectroChem) was employed as the counter electrode. SAHPO powder was synthesized according to a previously reported procedure^38^. PTFE powder (0.04 g) was added to 1.00 g of SAHPO powder, kneaded using a mortar and pestle, and then cold-rolled to a thickness of 250 μm using a laboratory rolling mill.

### Characterization

AC conductivity measurement of the composite electrolyte membrane was performed in atmospheric air using two Pt/C electrodes. Impedance spectra were recorded using an impedance analyzer (Solartron, SI 1260) and an electrochemical interface (Solartron, 1287) in the frequency range of 10–10^6^ Hz with an AC amplitude of 10 mV. The H_2_ and O_2_ permeabilities of the composite membrane were determined using two chambers separated by the membrane. Pure H_2_ or air was supplied to one chamber and N_2_ to the other at a flow rate of 10 mL min^−1^; the amount of H_2_ or O_2_ that entered the N_2_ chamber was monitored using gas chromatography (Varian, CP-2002) with a thermal conductivity detector. The total acid content of SAHPO was determined according to the method suggested by Boehm^29^ using NaOH as a reaction reagent. Elemental depth profiles were analyzed using Auger electron spectroscopy (Ulvac-Phi, SAM670) with a beam voltage of 10 kV and a current of 20 nA. Sputtering was accomplished with a 2 kV argon ion beam. The sputter rate was calibrated on SiO_2_ to be 4 nm min^−1^. Sputter depths reported in this work are given as the depth calibrated against the sputter rate of SiO_2_ for the same time and conditions.

### Electrochemical measurements

Electrochemical devices were fabricated by sandwiching the electrolyte membrane between two electrodes (projected surface area^39^ 0.5 cm^2^). LSV and CV measurements between the two electrodes were conducted in atmospheric air. The scan rate in these trials was set at 200 mV s^−1^, which provides a good balance for the redox potential and switching characteristic measurements. The 2nd CV curves were recorded as data, unless otherwise stated. Impedance spectra of the device were acquired at various bias voltages or at open-circuit voltages in the frequency range of 0.1–10^6^ Hz with an AC amplitude of 50 mV. Switching tests were performed by applying bias voltages of ±7 V to the device for 1 or 0.1 s. (It is technically difficult to obtain current signals with high accuracy within 0.1 s or less with the equipment used.) The overpotentials of a Ti powder electrode (Wako Chemicals) were analyzed in atmospheric air using the current interruption method, wherein a sharp change in voltage corresponds to ohmic loss, and a slow voltage change corresponds to non-ohmic polarization losses. In the present case, the Pt/C reference and counter electrodes were attached to the side of the electrolyte sample and the electrolyte surface, respectively.

## Additional Information

**How to cite this article**: Hibino, T. *et al*. Kinetically driven switching and memory phenomena at the interface between a proton-conductive electrolyte and a titanium electrode. *Sci. Rep.* **6**, 31691; doi: 10.1038/srep31691 (2016).

## Supplementary Material

Supplementary Information
