# Supplementary material for: Kinetically driven switching and memory phenomena at the interface between a proton-conductive electrolyte and a titanium electrode
Source: Sci Rep. 2016 Aug 16;6:31691. doi: 10.1038/srep31691 (PMC4985656; doi:10.1038/srep31691)

Supplementary Information

Kinetically driven switching and memory phenomena at the interface between a proton-conductive electrolyte and a titanium electrode

Takashi Hibino*, Kazuyo Kobayashi & Masahiro Nagao

Graduate School of Environmental Studies, Nagoya University, Nagoya 464-8601, Japan

*hibino@urban.env.nagoya-u.ac.jp

+these authors contributed equally to this work

Table S1. Physicochemical characteristics of a 250 μm thick SAHPO-PTFE composite membrane.

Conductivity [a]	Ohmic resistance [a]	Acid content	Gas penetration rate	
S cm^{-1}	Ω cm^2	mEq g^{-1}	mmol m^{-2} s^{-1}	
			H_2	O_2
5.92×10^{-4}	42.3	0.07	5.94	0.61

[a] These data were recorded in atmospheric air with a relative humidity of approximately 50%.

Figure S1. XRD patterns and SEM images of SAHPO and Ti before and after electrochemical measurements.

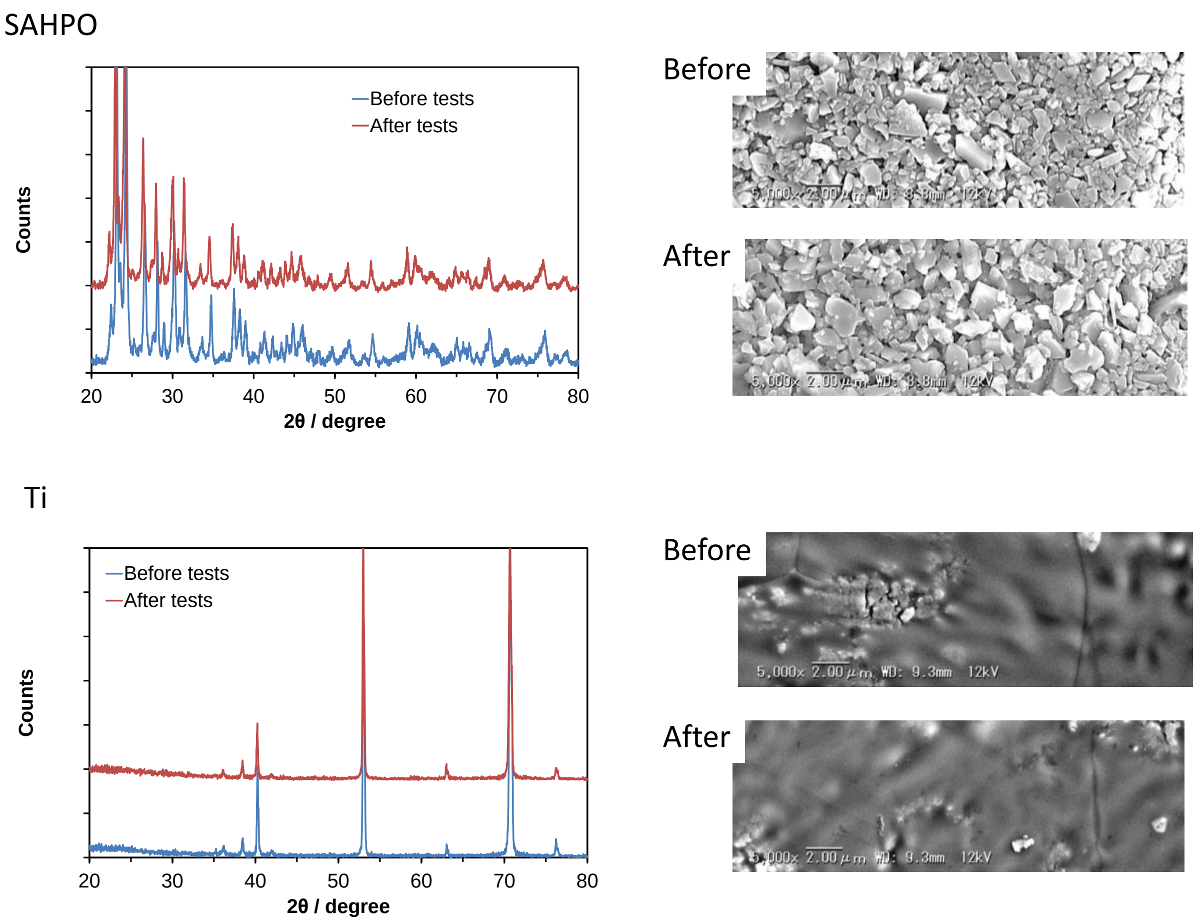

Figure S2. Impedance and bode plots for the device at open-circuit voltage after application of a bias voltage of -7 V (Step 1). The fitted values (black solid and dotted lines) are computed from an equivalent circuit model.

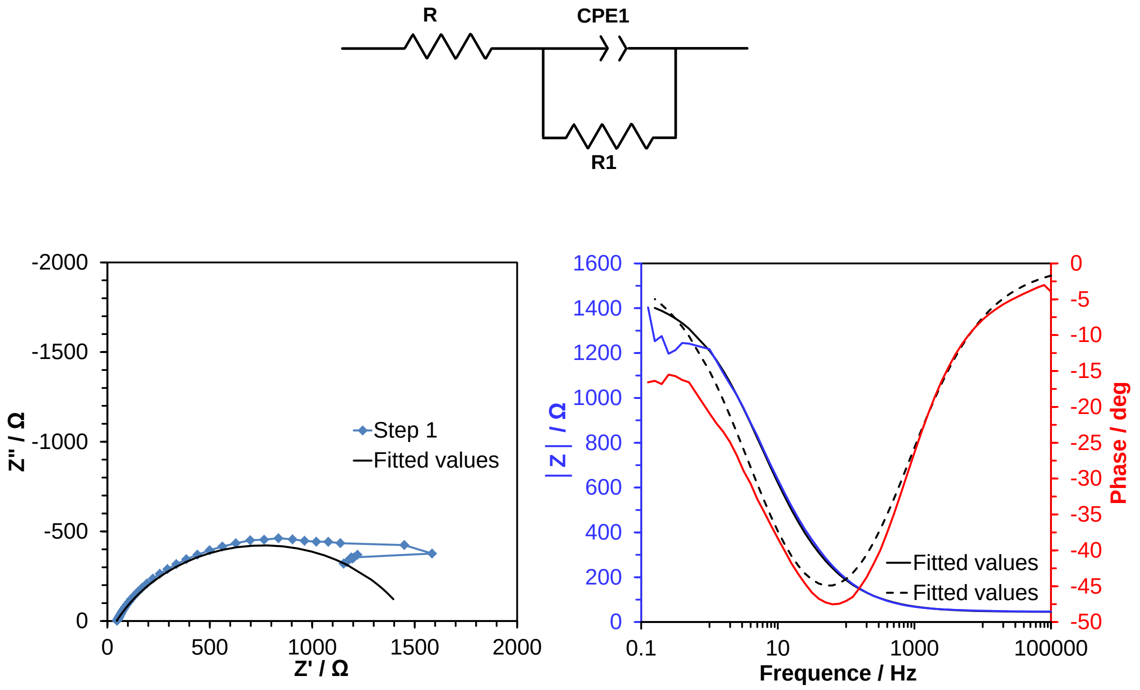

Figure S3. Impedance and bode plots for the device at open-circuit voltage after application of a bias voltage of +7 V (Step 2). The fitted values (black solid and dotted lines) are computed from an equivalent circuit model.

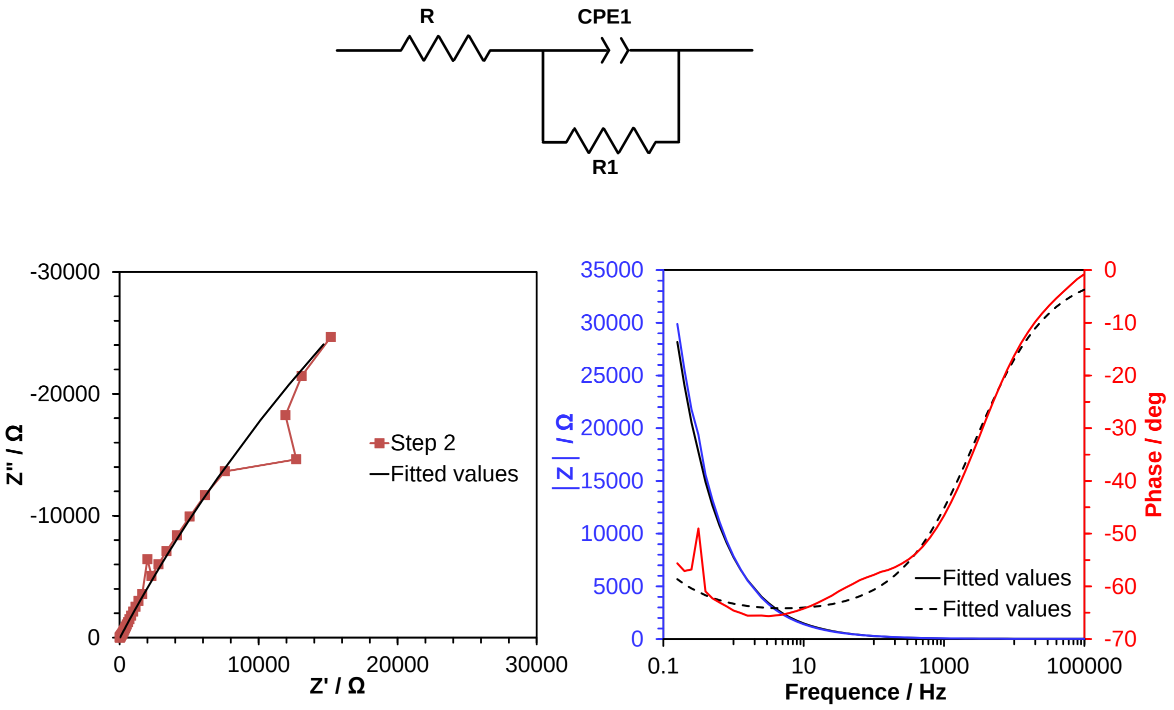

Figure S4. Auger depth profiles for the Au electrode before and after electrochemical measurements.

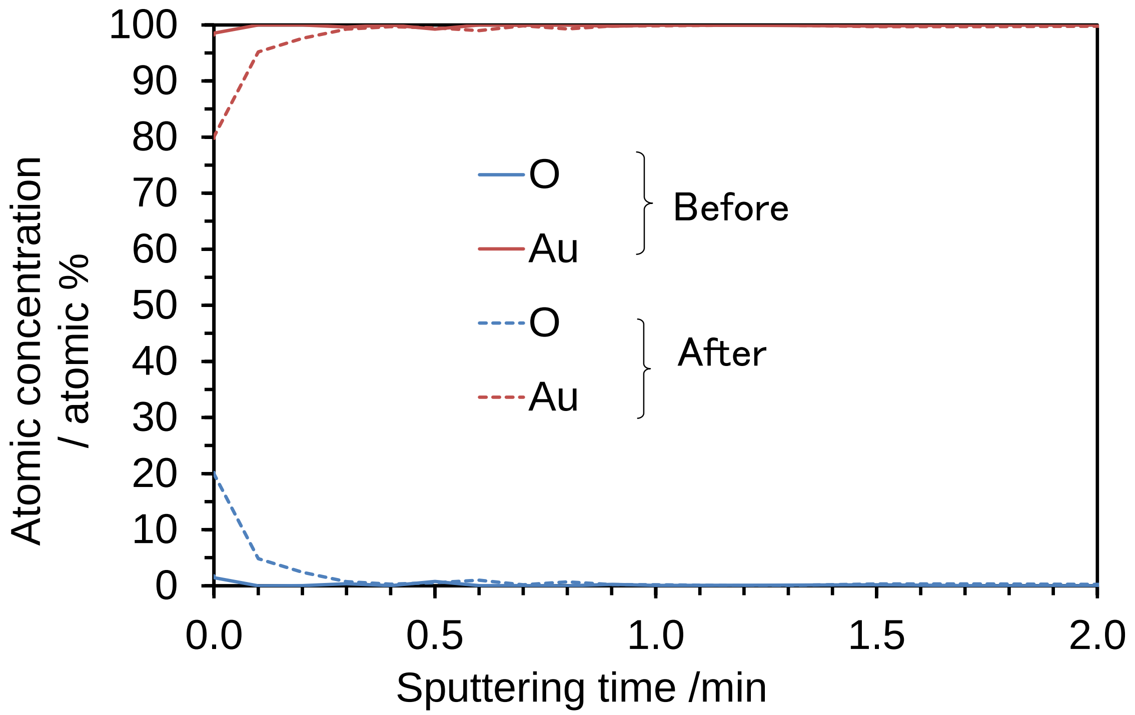

Supplement: Supplementary Information [file srep31691-s1.pdf]
